# Interactions of a pesticide/heavy metal mixture in marine bivalves: a transcriptomic assessment

**DOI:** 10.1186/1471-2164-12-195

**Published:** 2011-04-16

**Authors:** Francesco Dondero, Mohamed Banni, Alessandro Negri, Lara Boatti, Alessandro Dagnino, Aldo Viarengo

**Affiliations:** 1 Department of Environmental and Life Sciences, Università del Piemonte Orientale Amedeo Avogadro, 15121 Alessandria, Italy; 2 Laboratory of Biochemistry and Environmental Toxicology, ISA, Chott-Mariem, 4042, Sousse, Tunisia

## Abstract

**Background:**

Mixtures of chemicals present in aquatic environments may elicit toxicity due to additive or synergistic effects among the constituents or, vice versa, the adverse outcome may be reduced by antagonistic interactions. Deviations from additivity should be explained either by the perturbations of toxicokinetic parameters and/or chemical toxicodynamics. We addressed this important question in marine mussels exposed subchronically to a binary mixture made of two wide-spread pollutants: the heavy metal nickel and the organic phosphorus pesticide Chlorpyrifos. To this aim, we carried out in tissues of *Mytius galloprovincialis *(Lam) a systems approach based on the evaluation and integration of different disciplines, i.e. high throughput gene expression profiling, functional genomics, stress biomakers and toxicokinetics.

**Results:**

Cellular and tissue biomarkers, viz. digestive gland lysosomal membrane stability, lysosomal/cytosol volume ratio, neutral lipid content and gill acetylcholinesterase activity were, in general, altered by either the exposure to nickel and Chlorpyrifos. However, their joint action rendered (i) an overall decrease of the stress syndrome level, as evaluated through an expert system integrating biomarkers and (ii) statistically significant antagonistic deviations from the reference model systems to predict mixture toxicity. While toxicokinetic modeling did not explain mixture interactions, gene expression profiling and further Gene Ontology-based functional genomics analysis provided clues that the decrement of toxicity may arise from the development of specific toxicodynamics. Multivariate statistics of microarray data (238 genes in total, representing about 14% of the whole microarray catalogue) showed two separate patterns for the single chemicals: the one belonging to the heavy metal -135 differentially expressed genes (DEGs) was characterized by the modulation of transcript levels involved in nucleic acid metabolism, cell proliferation and lipid metabolic processes. Chlorpyrifos exposure (43 DEGs) yielded a molecular signature which was biased towards carbohydrate catabolism (indeed, chitin metabolism) and developmental processes. The exposure to the mixture (103 DEGs) elicited a composite complex profile which encompassed the core properties of the pesticide but also a relevant set of unique features. Finally, the relative mRNA abundance of twelve genes was followed by Q-PCR to either confirm or complement microarray data. These results, in general, were compatible with those from arrays and indeed confirmed the association of the relative abundance of two GM-2 ganglioside activator genes in the development of the hyperlipidosis syndrome observed in digestive gland lysosomes of single chemical exposed mussels.

**Conclusion:**

The transcriptomic assessment fitted with biological data to indicate the occurrence of different toxicodynamic events and, in general, a decrease of toxicity, driven by the mitigation or even abolition of lysosomal responses. Furthermore, our results emphasized the importance of the application of mechanistic approaches and the power of systems assessment to study toxicological responses in ecologically relevant organisms.

## Background

Pollutants are present in aquatic environments in the form of complex mixtures whose single compounds may be not toxic, *per se*, at the considered concentrations [1,2]. Although scientists generally have a good understanding of the toxicity of individual chemical pollutants, there is a great need to bridge the gap between our understanding of the toxic effects of exposure to individual xenobiotics and those effects from exposure to mixtures of such chemicals [3]. The toxic effects of mixtures are usually predicted from reference models based on non-interaction among the single chemicals, i.e. concentration addition [4] and response addition [5] have been established. Several studies dealing in particular with classical ecotoxicological endpoints such as mortality and reproduction, have suggested the validity of both models as a first screening to analyze the toxicity of mixtures [6]. However a considerable part of mixture data (20-40%) is not describable by such models, indicating a significant interaction among chemicals, leading to synergistic or antagonistic outcomes [2]. Even if limited to binary mixture assessments, recently improved mathematical models have been proposed to describe such deviations [7]. However, the great challenge still remains of predicting such occurrences and providing mechanistic explanations on mixture toxicity.

Mussels have been extensively used in biomonitoring projects through the application of a battery of physiological and cellular biological responses able to prove the occurrence of a stress syndrome and the biological risk associated with polluted environments [8-12]. Recently, furthermore, transcriptomics approaches have been successfully applied to these species to unveil the molecular mechanisms of adaptation to both natural and chemical stressors [13-15]. With the advent of the post-genomic and second generation sequencing era, ecotoxicologists have enthusiastically embraced trancriptomic profiling as a tool to assess exposure to environmental stressors [16]. However, gene expression may not represent a direct marker of functional responses as only gene products and metabolites do represent the final cellular effectors Recently, systems biology -a combination of high-throughput molecular disciplines- was proposed to improve the landscape of protective and non-protective responses occurring in cell/tissues of a given bioindicator species. This specific application is known as systems toxicology and is defined also as the study of perturbations of biological systems by chemicals and stressors as well as monitoring changes in molecular expression and conventional toxicological parameters [17]. A successful example is represented by the study of the non-model organism *L. rubellus*, from which was obtained a strong inference about the mechanistic effects of copper on earthworms [18].

A first attempt on marine bivalves was proposed by [19] who showed the benefit of the integration of biomarkers with gene expression changes to interpret the physiological status of specimens collected along a copper pollution gradient. Notwithstanding, that work was limited by the assessment of a few stress genes. More recently, our research group has developed a high density (1.7 K) cDNA array and used it to assess distinct molecular fingerprints in the tissues of mussels exposed either to model contaminants in laboratory conditions or collected from field sites challenged with different pollutant levels [15].

We wondered whether transcriptomics and, in particular, a systems toxicology assessment may effectively represent a valuable approach for studying the biological effects of a mixture of pollutants and specifically to predict the interactions among its members, as recently proposed [20]. Here we present results based on transcriptomics, physiological biomarkers (lysosomal responses) and toxicokinetics measurements obtained in the digestive gland tissue of the marine bivalve *Mytilus galloprovincialis *exposed to a binary mixture of chemicals for four days. The toxic agents were selected as dissimilarly-acting toxicants from a panel of priority environmental pollutants within the framework of the European Project NoMiRACLE [21]: Chlorpyrifos, a broad-spectrum organophosphate insecticide and nickel (Ni), an important heavy metal for its civil and industrial applications. Chlorpyrifos represents one of the most utilized phyto-pharmacological products in the world for both crop protection and pest control [22] It's present in the marine environment, even including biota, of the American coast since the early 1990's [22]. Like other organophosphorus compounds, its mode of action is mainly based on the inhibition of the acetylcholinesterase system, both in vertebrate and invertebrate models [22-24]. In addition, several studies identified putative neurodevelopmental mechanisms that are independent of cholinesterase inhibition [25-28]. CHP has been shown to interfere with different components of cell signalling [29,30] and to affect oxidative stress parameters in the developing brain, leading to shifts in expression and function of antioxidant genes [31,32]. On the other hand, nickel is considered a dangerous pollutant, in particular for the recognized carcinogenic activity probably related to the production of oxidative DNA damage and the inhibition of DNA repair activity. Furthermore, nickel can produce oxidative stress that depletes glutathione, activates Ap1, NF-kB and other oxidatively sensitive transcription factors. Nevertheless, exact knowledge of the molecular mechanisms of nickel toxicity and carcinogenicity is still limited [33]. Recently, a few studies reporting the ecotoxicological effects due to the joint Ni and Chlorpyrifos exposure in different model species were published as results of the NoMiRACLE project [34-37]. Here we report our original findings on mixture toxicity analysis using a systems toxicology approach in the marine mussel *Mytilus galloprovincialis*.

## Results

### Experimental design for mixture toxicity assessment

In a preliminary set of exposures, mussels were subjected to increasing concentrations of either Ni (0.01-15 mg/L) or the organophosphate pesticide Clorpyrifos-ethyl (CHP) (0.1-10 mg/L) for four days in aquaria (semi-static exposures). The effects on digestive gland lysosomal membrane stability (LMS) were evaluated on frozen cryostat sections and these data were used to calculate toxicity endpoints (EC values) through a log-logistic regression (Table 1, see Additional file 1). LMS represents a well known biomarker of stress which is considered a good predictor of the physiological status of mollusks [38,39]. Therefore, this parameter was used as the guide biomarker in the mixture toxicity assessment. To this end, we implemented a fixed experimental design encompassing three different nominal dose levels of the single chemicals -0.25; 0.5; 1.0 Toxic Unit (TU) (Table 1)- and two equitoxic mixtures at nominal 0.5 TU or 1.0 TU. According to the Concentration Addition (CA) model proposed by [4] these toxic levels are obtained through the combination of, respectively, ^Ni^EC12.5 plus ^Clp^EC12.5 or ^Ni^EC25 plus ^Clp^EC25.

**Table 1:** Toxicity endpoints

	EC12.5 (0.25 TU)	EC25 (0.5 TU)	EC50 (1.0 TU)
Ni (mg/L)	0.022	0.135	0.770

Clorpyrifos (mg/L)	0.300	0.610	4.500

Shown are Ni and Chorpyrifos effective concentrations (EC) and equivalent Toxic Units (TU) for Lysosomal membrane stability (LMS) obtained from the dose finding experiment (see Additional file 1). The dimensionless toxic unit (TUi) quantifies the relative contribution to toxicity of the individual chemical i in a mixture of n chemicals. Conventionally, a TU is defined as the actual exposure concentration of a chemical divided by its EC50 (the median effect concentration).

### Physiological responses in bivalves exposed to Ni/Chlorpyrifos mixture showed antagonistic deviations from the reference model systems

**Figure 1:**
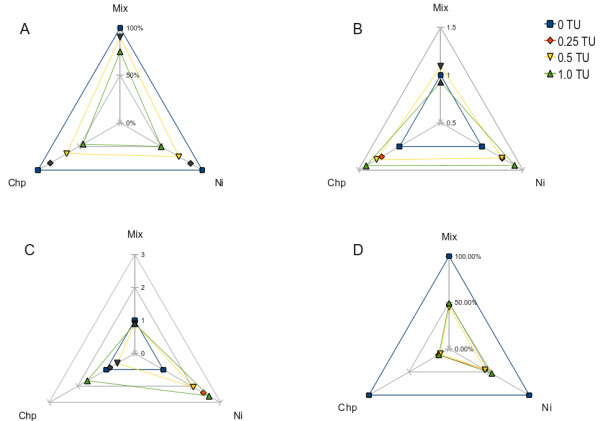
**Biological effects of Ni and Chlorpyrifos in the form of single chemicals and mixture**. The wire charts depict the biomarker outcomes obtained in tissues of animals challenged with Ni, Chlorpyrifos and their mixtures at nominal equitoxic levels for lysosomal membrane stability. A. Digestive gland lysosomal membrane stability (LMS) determined as residual N-acetyl-B-hexosaminidase activity (% labilization time) in cryostat section; 100% activity was obtained in control samples at 20 min of incubation in acidic-citrate buffer. B. Digestive gland lysosome/cytoplasm ratio in same sections. C. Digestive gland lysosomal lipid accumulation (fold change) evaluated by red oil staining and digital image analysis in cryostat section. D. Gill acetylcholinesterase residual activity (%) evaluated in S10 supernatant; 100% activity was calculated in control samples as 60.3 μmoles/min/mg. Legend (blue square, 0 TU, control; red diamond, 0.25 TU; yellow inverted triangle, 0.50 TU; green triangle, 1 TU). Symbols marked with a color other than grey depict a difference with respect to control (Mann Whitney U-test, p < 0.05).

**Figure 2:**
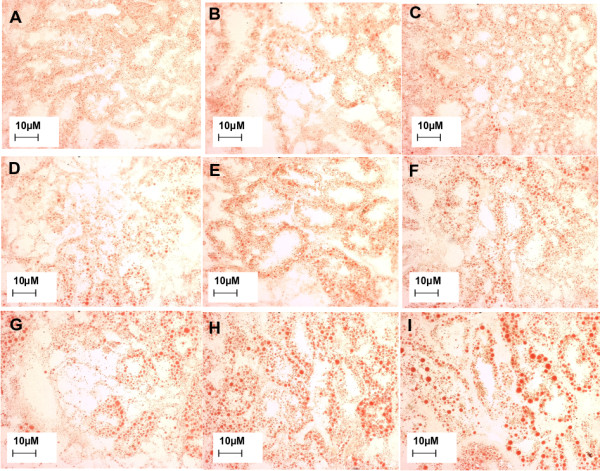
**Oil Red-O staining of lipids**. cryostat sections of frozen mussel digestive gland were stained with the lysochrome Oil Red-O dye which is able to color neutral triglycerides and glycolipids. Panel A, control reference (vehicle treated); B, mixture 0.5 TU; C, mixture 1 TU; D, Chlorpyrifos 0.25 TU; E, Chlorpyrifos 0.5 TU; F, Chlorpyrifos 1 TU; G, Ni 0.25 TU; H, Ni 0.5 TU; I, Ni, 1 TU (see Table 1 for details on chemical concentrations).

The biological effects of exposure to the single chemicals and mixtures were evaluated through a battery of biological endpoints encompassing lysosomal responses in the digestive gland (LMS; lysosome/cytoplasm ratio; lipid accumulation) and acetylcholinesterase activity in the gills (Figure 1). As expected, the LMS assay showed a clear dose-response trend with a decrement of the hexosaminidase activity latency along with the increase of pollutant concentrations (Figure 1, panel A). An increased lysosomal/cytoplasm ratio was also observed (Figure 1, panel B). The lipid content rose dramatically at all Ni doses while the biocide elicited a significant response only at the highest tested concentration (Figure 1, panel C; Figure 2). Acetylcholinesterase activity was evaluated in the gills of bivalves as a specific biomarker of exposure to the organophosphate pesticide. About 80% inhibition and a typical threshold effect was ascertained in the case of Chlorpyrifos exposure. However, a significant effect and a similar trend was also observed in tissues from Ni exposures (Figure 1, panel D). Our results from mixtures indicated that n-acetyl-beta-hexosaminidase latency times in destabilized lysosome membranes were higher than the expected values calculated according to either the Concentration Addition or the Independent Action (IA) mixture toxicity reference system models (Table 2). This antagonistic outcome was also confirmed for the other lysosomal responses evaluated in the digestive tissue, viz. organelle enlargement, lipid accumulation and the inhibition of the acetylcholinesterase activity in gills (Table 2).

**Table 2:** Statistical testing of antagonistic interactions

	LMS	LYS/CYT	NL	AchE
n	49	45	158	72

***Fit of CA***				

R^2	0.73	0.64	0.74	0.77

P-value	5.59E-012	2.96E-009	2.48E-044	1.01E-020

CA vs A				

R^2	0.79	0.88	0.89	0.85

Chi-test	1.27E-003	2.30E-025	2.64E-030	4.05E-008

A vs DL				

R^2	0.78	0.88	0.89	0.85

Chi-test	0.30	0.89	0.94	0.23

***Fit of IA***				

R^2	0.75	0.78	0.74	0.64

P-value	8.52E-013	5.25E-015	3.06E-043	4.05E-014

IA vs A				

R^2	0.78	0.85	0.79	0.85

Chi-test	1.10E-002	9.63E-011	1.17E-009	4.81E-015

A vs DL				

R^2	0.78	0.85	0.74	0.84

Chi-test	0.63	1	1	1

The Mixtox software [7] was utilized to evaluate antagonistic interactions in binary mixtures fitting experimental data to both the CA and IA reference models and their deviation models describing antagonistic interactions, either through the whole dose range (A) or varying across the dose levels (DL). R^2 represents the amount of the total data variance explained by each tested model. P-value represents the significance of data regression against the "null hypothesis" of no relationship between increasing doses and effects for the whole dataset. Chi-test represents the p-value of the Chi-square distribution test to assess if a significantly better fit is achieved by going to the next level of the nested models (i.e. CA vs. A and A vs. DL). The A models always provided a better description of the data than their respective parent CA or IA models, while there were no significant improvements by using models explaining a dose level dependent antagonism (DL).

**Figure 3:**
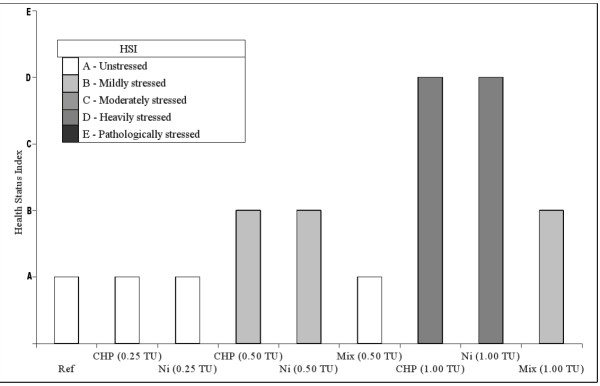
**Mussel Health Status Index (HSI)**. The physiological state of mussels was classified according to the outcome of a rule-based algorithm (expert-system) integrating the responses obtained from the different biomarkers [38]. Legend: ref, control reference (vehicle-treated) mussels; Ni, nickel- exposed mussels; CHP, Chlorpyrifos-exposed mussels; Mix, mixture exposed mussels. (Details on chemical concentrations are reported in Table 1).

The results obtained from the battery of physiological markers were then integrated using an expert system able to rank the physiological status of bivalves in a five-level scale ranging from un-stressed to pathological [38]. The output provided a clear indication that (i) in mussels the stress level rose along with the concentration of the two chemicals and (ii) animals exposed to mixtures presented a better health status index than those exposed to single chemicals at the same nominal degree of toxicity (Figure 3). We also studied the ability of mussels to eliminate toxicants after the exposure fitting chemical data into a toxicokinetics model, thus deriving the elimination constant for each condition (Table 3).

**Table 3:** Toxicokinetic parameters

	**k (d**^**-1**^**)**	**R**^**2**^
Ni	0.0076	0.87

^mix^Ni	0.0012	0.93

CHP	0.0470	0.95

^mix^CHP	0.0440	0.75

Shown are: k, the elimination constant for each tested condition empirically evaluated from model fitting. R^2^, variance explained by the model. All conditions were tested at 1 nominal TU (see Table 1 for details on concentrations).

### *Gene expression profiling in pollutant-exposed organisms*

**Figure 4:**
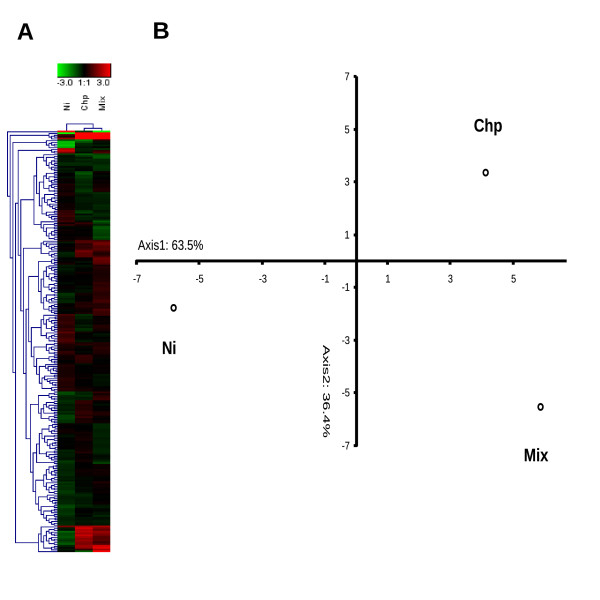
**Multivariate analysis of gene expression data**. Panel A: cluster analysis (Heat Map) (Euclidian distance, complete linkage algorithm. Panel B: Principal component analysis (Pearson correlation). For these analyses it was considered a set of 238 differentially-expressed genes in at least one condition.

To obtain more clues on mixture toxicity mechanisms, we carried out dual-color hybridisation microarray analysis by means of a 1.7 K cDNA array [15] in the mussel digestive gland (see Additional file 2). These data were then integrated with a real-time quantitative PCR (Q-PCR) analysis of selected genes (Table 4 ). Gene expression profiles were evaluated in the digestive tissue of bivalves exposed to 1 nominal TU (Table 1) as single chemicals and mixture. A total of 238 differentially expressed genes (DEGs) were identified in at least one condition by means of moderated Baesyan statistics (see Additional file 2; Figure 4). The largest molecular response was observed in the case of Ni displaying up to 135 DEGs of which 64% were down-regulated. A different trend was instead reported for Chlorpyrifos, with the least amount of DEGs, i.e. 43 of which 65% up-regulated. The effects of the mixture exposure rendered 103 DEGs, almost equally represented by up and down regulated features (55% and 45% respectively). The largest part of them (61, 59%) were unique genes, not shared with neither Ni nor Chlorpyrifos (Table 5). Multivariate statistical procedures were carried out on the whole set of 238 genes by means of hierarchical clustering and Principal Component Analysis. These analyses clearly rendered two different gene expression profiles in tissues of Ni- or Clorpyrifos-exposed animals and a higher correlation between the mixture and the pesticide (Figure 4).

**Table 4:** Real time Q-PCR analysis: comparison with microarray data

				Ni			Chlorpyrifos			Mix	
**Array_ID**	**EMBL ID**	**Description**	**Q-PCR**	**M**	**B**	**Q-PCR**	**M**	**B**	**Q-PCR**	**M**	**B**

Myt01-016C08	AJ625847	metallothionein isoform mt-10b	2.8*	1.46	11.5	-0.3	-0.1	-6.3	1.8*	0.9	4.4

N.A.	AY566247	metallothionein isoform mt-20-IV	0.6	N.A.	N.A.	-0.1	N.A.	N.A.	0.0	N.A.	N.A.

Myt01-009A12	AJ624405	gm2 ganglioside activator protein	13.6*	2.63	7.0	-0.7	-0.3	-5.4	-7.6*	0.5	-1.3

Myt01-009E10	AJ624495	gm2 ganglioside activator protein	-4.4*	-0.87	9.7	5.6*	-0.6	-5.0	6.7*	0.4	0.1

Myt01-016D06	AJ625863	apolipophorin precursor	0.8*	0.56	5.1	0.3	-0.1	-6.1	-0.6	-0.0	-6.4

Myt01-015B01	AJ625569	chitinase	1.7	-0.08	-7.2	6.4*	1.9	12.2	6.6*	1.7	4.7

Myt01-007F12	AJ624093	chitinase	1.5	-0.41	2.3	5.4*	1.9	9.9	5.6*	2.0	0.4

Myt01-010C02	AJ624637	chitinase	0.9	-0.52	3.6	5.7*	1.6	4.5	6.0*	1.7	1.8

Myt01-012D02	AJ625051	chitinase 1	0.4	-0.8	7.5	5.1*	3.2	11.5	6.2*	2.0	0.3

Myt01-004H06	AJ623463	beta-n-acetyl-hexosaminidase	-0.1	0.03	-7.5	-0.4	-0.3	-6.1	2.9*	1.1	-5.7

Myt01-012F08	AJ625116	actin	-0.0	-0.05	-6.9	0.4	0.1	-6.3	0.6*	0.5	2.7

Myt01-013C11	AJ625243	p-53 like	0.1	-0.11	-7.2	-0.4	0.3	-6.3	-0.1	-0.6	-3.9

Match (absolute; relative)				8; 0.73			10; 0.91			9; 0.82	

Shown are: Array_ID, unique identifier; EMBL ID, EMBL database gene identifier; Description, B2GO annotation; Q-PCR, log2 mean relative expression level, (to non exposed reference mussels) obtained by means of Q-PCR analysis; M, relative expression level obtained by means of microarray analysis; B, Bayes Log Odd, where B > 0 denotes a statistically significant differentially expressed gene in microarray analysis.

Match indicates the absolute and relative frequency of accordance between microarray and Q-PCR analysis for each class of treatments. A match was assigned whether (i) a pairwise comparison showed the same statistically significant (positive or negative) expression trend or (ii) independently on the expression trend, when both analysis outcomes were not statistically significant. The AY566247 gene was not present in the microarray and therefore it was not considered for this analysis.

Relative expression levels were geometrically normalized against a 18 rRNA ribosomal target and an invariant microarray gene, alkaline phosphatase. * (p < 0.05; n = 4), threshold cycle reallocation randomization test according to [74]. N.A., not available

**Table 5:** Common gene frequency

condition	match	same trend	opposite trend
CHP/Mix	15% (15)	73% (11)	27% (4)

Ni/Mix	19% (19)	26% (5)	74% (14)

Ni/CHP	6.3% (15)	33% (5)	67% (10)

Ni/CHP/Mix	3.3% (8)	-	-

Unique Mix	59% (61)	-	-

Unique Mix including opposite	77% (79)	-	-

Shown are raw frequencies and -on brackets- absolute gene numbers in common between each single chemical and the mixture, or unique in the mixture ("match"); raw frequencies and absolute common gene number with "same" and "opposite" trend between each single chemical and the mixture Frequencies in first column were calculated on the basis of the total gene number observed in the mixture (103 genes). In the other cases, these were computed with respect to the total number of DEGs (238)

Real-time quantitative PCR was carried out to confirm microarray data and refine the relative expression levels of a selection of paralogue genes such as four chitinases, two metallothionein genes (mt10, mt20) and two ganglioside GM2 activator proteins, the latter involved in lysosomal lipid metabolism. Moreover, another two genes implicated in lipid metabolic processes were selected to confirm the relevance of this pathway in exposed bivalves: hexosaminidase and apolipophorin precursor. An actin variant and the p53-like protein gene were also included in this survey. Microarray and Q-PCR analysis showed consistent outcomes in 27 out 33 (82%) comparisons made on the three classes of toxic treatments, but chitinase and GM2-AP genes, in some cases, could not be confirmed (Table 4). Considering the high degree of identity of such sequences (data not shown), it is likely that microarray probes could not provide a reliable assessment and therefore Q-PCR outcome was further considered for the discussion of data. In mixture exposed tissues, Q-PCR analysis could also confirm the up-regulation of the hexosaminidase gene which from microarray showed a positive, but not statistically significant, expression level value.

### Functional genomics analysis

**Figure 5:**
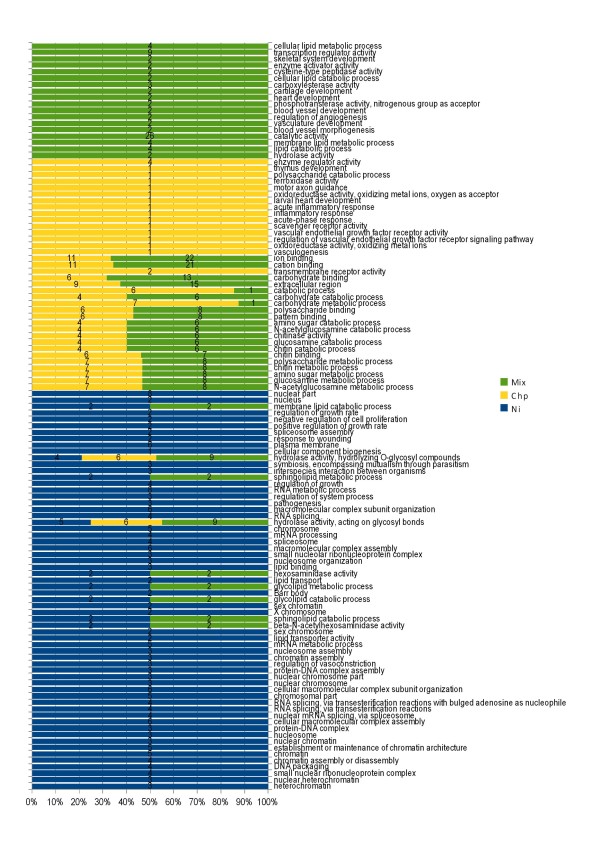
**Qualitative analysis of gene expression**. The bar chart depicts over-represented GO terms obtained from the list of differentially-expressed genes obtained in single chemical and mixture exposed samples. Enriched terms were selected through hypergeometric statistics comparing the GO term distribution in each gene list and the whole set of mussel sequences printed onto the array (Fisher exact test, p < 0.05) [75]. Bar length represents the relative frequency (%) of a GO term in each analyzed condition. Shown is also the number of genes associated with each GO term.

**Figure 6:**
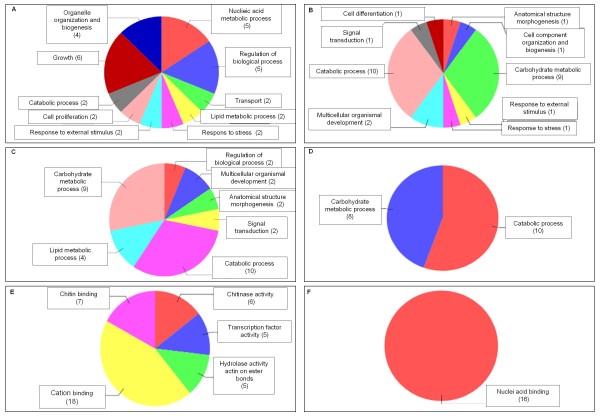
**Functional genomics analysis**. Enriched GO-terms (biological process) -obtained comparing the GO term distribution in each gene list and the whole set of mussel sequences printed onto the array (Fisher exact test, p < 0.05)- were used to generate multi-level pie charts showing the lowest node in each branch of the GO trees (Panel A, nickel; B, Chlorpyrifos; C, Mixture). The other charts display overrepresented GO terms obtained comparing the GO term distribution in mixture vs each single chemical treatment (Fisher exact test, p < 0.05). Legend: Panel D and E, Mixture vs Ni, biological process and molecular functions, respectively; Panel F, Mixture vs Chlorpyrifos, molecular functions (no results in the biological process clade were obtained).

The Blast2GO platform, a bioinformatic tool which statistically assigns Gene Ontology (GO) terms to unknown genes based on sequence information and a rule-algorithm [40] was utilized for the functional annotation of the *Mytilus galloprovincialis *transcriptome represented on the array. 1673 non-redundant mussel sequences obtained by tissue specific unbiased cDNA libraries and deposited into the EMBL database were considered for the analysis. 880 sequences showed no detectable homolog in other organisms and therefore are orphan genes. Another 63 sequences showed at least a Blast-X hit, but no GO terms were then associated. Finally, 873 (52.2%) mussel sequences were putatively annotated using GO terms obtained from the first 20 Blast-X hits [41] or from protein domains obtained from the InterPro database [42]. Several basic biological processes were represented on the array, such as protein modification process, lipid catabolic process, protein amino acid phosphorylation, response to unfolded protein, cellular metabolic process, transcription, growth, etc. 62 sequences were associated with the GO terms stress response. The annotation table was further used to implement a functional genomics analysis of gene expression data based on the enrichment of GO terms associated with differentially expressed genes (DEGs). Our results showed that GO terms were specifically associated with single chemicals. Moreover, the mixture showed a relevant amount of unique GO terms and a larger overlap with Chlorpyrifos (Figure 5-6, see Additional file 3 and 4).

## Discussion

The basic concept for the description of a toxicological action of components of a mixture is based on the principle of non-interaction, which means that chemicals in the mixture do not affect the toxicity potency of one another or each other's probability to exert a toxic effect [43]. Recent works focusing on the cumulative toxicity of Ni and CHP reported the occurrence of interactive effects at different levels of biological organization. An antagonistic interaction between the two chemicals on the locomotor activity of the zebrafish larvae was reported [34], while authors in [35] suggested a synergistic effect on the survival of the ground beetle *Pterostichus oblongopunctatus *indicating species-specific responses. In mussels, metabolomic profiling of digestive gland tissues obtained from animals exposed to Ni and CHP was compatible with a reduction in toxicity [36], arguing, therefore, antagonistic interactions. In the current study, we present for the first time in mussels, a systems biology assessment integrating the toxicokinetics and toxicodynamics of these chemicals and their mixture, thereby obtaining clues on the molecular mechanisms as the basis of pollutants interactions in mussel tissues.

### Ni and Chlorpyrifos elicited similar biological responses but distinct molecular fingerprints giving rise to a complex transcriptional profile in mixture

The exposure of marine bivalves to two different chemicals and their mixture determined biological responses with discrete quantitative levels through which it was possible to rank the health status of the organisms according to their stress syndrome. According to the biomarker expert system outcome, animals exposed to the mixture exhibited a better health status compared to those exposed to single chemicals at the same nominal toxic dose (Figure 3). A particular evidence it was the suppressive effect of Chlorpyrifos over the hyperlipidosis syndrome diagnosed in digestive gland lysosomes of Ni exposed mussels (Figure 2). However, the occurrence of deviations from the common mixture toxicity reference models based on non-interaction was confirmed for all biomarkers, testing the significance through maximum likelihood analysis (Table 2).

To get clues on interactions between heavy metal and pesticide toxicity, we first looked at the toxicokinetics but it did not provide an explanation for the attenuation in toxicity observed in mixture-exposed animals. In fact, looking at the parameters obtained by fitting the data into a kinetic model, the elimination constant (k) of CHP in the absence/presence of the heavy metal is almost identical, indicating no changes in the metabolism of the biocide. Moreover, the elimination constant of Ni indicated an even lower degree of detoxification when CHP was jointly administered to mussels (Table 3). We conclude, therefore, that the decrease in toxicity observed at a biological level could not arise from interaction at a toxicokenetic level as reported by [37] for the soil-dwelling collembolum *Folsomia candida*.

As it is well known that the mussel digestive gland is the most active metabolic organ [44] suitable for ecotoxicogenomic profiling [19,15], we, therefore, focused our attention to this tissue. Starting from nominal equitoxic effects on lysosomal membrane stability (Table 1), we carried out transcriptomic profiling using the 1.7 K cDNA chip (see Additional file 2; Figure 4) and further gene ontology-based functional genomics analysis (Figure 5-6, see Additional file 3). These analyses provided a picture of the biological processes and toxicodynamics implicated in the response to the two pollutants and their mixture. In the case of single exposures, our results pointed out two distinct gene expression profiles (Figure 4). Divergent gene expression profiles have already been reported in another model species, i.e. differentiating PC12 mouse brain cells challenged with the same chemicals [29,45,46]. In the case of the mixture, a complex pattern was observed which accounted for some genes inherited from the single chemicals -in particular Chlorpyrifos- showing the same relative expression trend; a set of unique sequences and a fraction of genes showing an opposed relative expression trend with respect to that observed in single chemical exposures (Table 5).

### Functional genomics of single chemical exposures

Ni exposure increased the expression level of the metallothionein mt10 gene, while negligible effects were observed for the cognate sequence mt20 (Table 4). In addition, transcriptomic profiling, by means of microarray analysis, allowed the detection of several genes which appear to be involved in epigenetic processes, as already reported for different heavy metals and also other environmental perturbations [19,47]. In fact, among the significant molecular features observed exclusively in Ni-treated organisms there are those putatively involved in spliceosome assembly and the establishment of chromatin architecture, as outlined by the Blast2GO analysis (Figure 5; see Additional file 3). Ni elicited the specific down-regulation of four different, small, nuclear, ribonucleoprotein which are homologous with components of the Sm core complex [48] and a poly-A-binding cytoplasmic 4 like-protein, involved in the half-life regulation of labile mRNAs [49]. With the same trend of down-regulation, we found also histone h3, histone h2a, histone aminotransferase 1 and a heterochromatin protein 1 family member which, in different organisms, is reported to bind histone H3 methylated at Lys 9, thus leading to gene silencing through heterochromatin promotion [50].

Moreover, Ni modulated several genes involved in the regulation of biological processes such as proliferation, growth and apoptosis (Figure 6; see Additional file 4). Examples are given by a b-cell translocation gene 1 homologue (upregulated) with putative anti-proliferative functions [51]; prohibitin (downregulated) which is an ubiquitous protein with a controversial role in cell proliferation processes [52] and biogenesis of mitochondria [53]; a (downregulated) defender-against-cell-death (dad-1)-like gene, identified as a negative regulator of programmed cell death in mammalian cells[54] and with a role in tissue regeneration in the marine scallop *Argopecten irradians *[55]. Finally, Ni exposure elicited the modulation of genes involved in lipid metabolism with two genes whose expression was up-regulated: an apolipophorin putatively involved in the transport of lipids to hemolymph in insects [56] -whose differential expression was also confirmed by Q-PCR (Table 4)- and a fatty acid-binding protein [GeneBank:AJ624395] with a role in the proliferation process [57]. Still concerned with lipid metabolism are two non-allelic variants of the GM2 ganglioside activator protein (GM2-AP) [GeneBank:AJ624405 and GeneBank:AJ624495] whose expression levels were dramatically up- and down-regulated, respectively (Table 4). GM2-AP are ubiquitous lysosomal proteins [58] which, in mammals, act as substrate-specific co-factors of β-hexosaminidase-A for the hydrolysis of GM2 ganglioside, a class of glycolipids positive to oil-red staining. Defects on both lysosomal β-hexosaminidase A and GM2-AP result in a fatal syndrome characterized by a hyper-accumulation of glycolipids in neuronal cells [59]. These two genes could represent the candidates for the strong increase of lipid accumulation in the digestive gland cells of mussels (Figure 1, panel C; Figure 2). The presence of two differentially expressed GM2-AP variants might represent a transcriptional mechanism to regulate lysosomal hexosaminidase activity, according to the physiological requirements of the organism. To this aim, it is important to point out that different GM2-AP expression patterns corresponded to different lipid disorder levels (Figure 1, panel C; Figure 2). The huge relative expression levels revealed by Q-PCR would suggest a typical switch-on/off transcriptional regulation.

Mussels exposed to Chlorpyrifos exhibited a marked decrease in acetylcholinesterase activity in the gills, independently of the concentration tested (Figure 1, panel D) and a similar effect was also previously demonstrated in the digestive gland by [36]. Acetylcholinesterase inhibition is a well-known biomarker of exposure to organophosphate pesticides in several organisms. However, in marine invertebrates, heavy metals may affect such enzymatic activity too [12], and, in fact, we herein report the suppressive effect of Ni (Figure 1, panel D). In what concerns the transcriptomic assessment, the most relevant biological process involved in response to Clorpyrifos was represented by carbohydrate catabolic process, in particular that related to chitinase activity (Figure 5). In fact, up to 5 different highly homologous chitinase genes were found hugely up-regulated from microarray analysis. TaqMan assays set up for four of these sequences confirmed the relative expression levels (Table 4). It is difficult to speculate on the biological meaning of such findings; however, in mussels and other marine invertebrates, chitinases play a role in digestion [60] and moreover, in hemocytes, they participate in the innate immune response [60,61]. Finally, GO enrichment analysis identified a series of statistically over-represented developmental and differentiation processes, even if these were related to the down-regulation of a single myosinase-like-gene.

### Functional genomics of mixture toxicity

Very little is known about the relationships between mixture toxicity and gene expression changes. Some studies reported that the transcriptional patterns found in mixture-exposed samples were largely inherited from the single chemicals, suggesting additive or weak interactive effects at a molecular level [62,63]. Conversely, other works reported much more important interactions so that exposure to a mixture determines -along with overlapping genes- a relevant number of sequences whose expression is exclusively modulated in this condition. These findings were observed in different biological models -rat, fish and crustacean- and for different types of chemical mixtures, i.e. binary, ternary, similarly- and dissimilarly-acting chemicals [64-66]. In our study, multivariate analysis of gene expression patterns showed that the molecular signature observed in mixture exposed samples is biased towards the effects of the pesticide (Figure 4). A similar evidence emerged also from the comparison of functional gene annotations in which GO terms such as carbohydrate catabolic process, multicellular organismal development, anatomical structure morphogenesis, signal transduction were shared with the pesticide. Nevertheless, the exposure to the mixture elicited the modulation of several unique genes giving rise to a molecular fingerprint which appears to be characterized by at least two original features: catalytic and DNA-binding (including transcription factor) activity (Figure 5, see Additional file 3). The direct comparison of GO term distributions demonstrated that these attributes were driven neither by Ni nor Chlorpyrifos (Figure 5-6). This kind of composite signature bearing common and unique features is consistent with that found by our research group in mussels exposed to a binary mixture of neonicotinoids insecticides [67]. Also Vandenbrouck and coworkers [65], studying transcriptomic effects of binary mixtures (nickel with other heavy metals) in the freshwater cladoceron *Daphnia magna*, found affected genes and pathways which were exclusive of the mixture exposure. More recently, another research group investigated in the worm *Caenorhabditis elegans *the joint effects of Chlorpyrifos and another organic phosphorus compound, diazinon, by means of gene expression techniques. They still concluded their work stating that the effect of a mix of low doses of the two biocides is not a summed effect of the single components, but at the same time, the similarities in the evoked pathways indicate the regulation of similar responses [68]. In general, these findings are in accordance with ours and therefore the presence of common and original responses seem to represent a common rule. We, however, identified one additional feature. In the present study, the three toxic treatments modulated the expression of some genes involved in lipid metabolism. This process was even significantly over-represented in Ni and mixture exposed tissues (Figure 5-6, see Additional file 3 and 4). In compliance with Vinuela and coworkers [68], not all transcripts of this group were identical among the three treatments (see Additional files 3, 4), except the two GM2-AP. These two transcripts, however, showed diametrically opposed expression levels and very huge fold change which cannot be explained simply by means of an additivity model (Table 4). This finding seriously poses for the implication of similar pathways but with a different biological meaning and in fact the outcome of lysosomal biomarkers was markedly different (Figure 1). Lipid metabolism was not a single case because these two molecular signatures were also characterized by a certain number of (dissimilar) genes with a possible role in apoptosis and cell proliferation (for the mixture: gadd45, caspase7, translationally controlled tumor protein (tctp) [GeneBank:AJ624761]; for Ni: b-cell translocation gene-1 homologue, prohibitin, dad-1), which considering their putative role in other model species, might be in contrast at a functional level, i.e. pro-apoptotic and pro-proliferative, respectively. However, this hypothesis remains open and requires further investigations, as we did not evaluated specific functional assays for such processes.

## Conclusions

We presented an analysis and comparison of the biological responses elicited by the exposure of marine mussels to two toxicants and a combination of both using a systems toxicology approach. Our results demonstrated the occurrence of interactive effects giving rise to an unpredicted response and finally to a decrease of toxicity. We identified and confirmed the core features of mixtures' gene expression profiles and highlighted novel developments. Furthermore, our findings underlined the fallacy of the "non-interaction" criterion usually applied to mixture toxicity prediction. This was particularly evident at a molecular level such as gene expression, thus putting a serious concern in the adoption of conventional mixture toxicity reference models in ecotoxicological surveys and risk assessment procedures.

This work demonstrated also that an integrated approach made of of transcriptomics, functional genomics, cellular and histological biomarkers can provide clues on complex biological responses, making links between different levels of biological organization, as we obtained in the case of lipid metabolism genes and lysosomal hyperlipidosis in the digestive gland.

Future implementation and development of massive sequencing applications and high density microarrays will fill the gap of genomic/transcriptomic information which is actually the major limitation in the use of *Mytilus galloprovincialis *as a model organism, thus providing more robust assessments into an ecologically relevant species ubiquitously present along the coastal marine environments.

## Methods

### Chemicals

Nickel was used in the form of chloride salt, obtained from Sigma Aldrich. Chlorpyrifos-ethyl was obtained from India Industrie Chimiche SPA (Padova, Italy). All other reagents were of analytical grade or "Chromasolv" grade for chemical analyses if not otherwise stated.

### Mussel exposures

For the range-finding tests, specimens of *Mytilus galloprovincialis *(5-6 cm length) were taken from a mussel farm in Cesenatico, North-East-Italy during March 2005, and transferred to aquaria with re-circulating aerated seawater, at 16°C collected offshore, at a density of 1 animal/L. After an acclimatizing period of 6 days, mussels were divided in groups of 5 vessels per 30 individuals each (150 mussels per condition) and further used as control reference or subjected to semi-static exposure to chemicals. Each vessel represented a parallel replicated experiment. Chemicals were administered every day, together with a commercial algal preparation (Liquifry, Interpret Ltd., Dorking, Surrey, UK) and seawater renewed every two days. Nickel was administered as the chloride salt (NiCl_2_) from a concentrated stock solution (5000 X), while Chlorpyrifos was diluted in dimethylsulphoxide (DMSO) and added at the desired concentration from concentrated stock solutions (5000 X). DMSO was also added to a control seawater and Ni-exposed mussels at the concentration of 0.02%. In all experiments female individuals, screened by microscopic inspection of gonads, were used for subsequent analyses.

### Dose range finding

Mussels (40 individuals per condition) were exposed for 4 days to nickel and Chlorpyrifos respectively in the range of 0.01-15 mg/L and 0.1-10 mg/L. Digestive gland cryostat sections were further scored for lysosomal membrane stability (LSM). Control groups of vehicle-treated mussels were kept in the same conditions as the ones exposed to the chemical.

### Mixture toxicity experimental design

For mixture toxicity analysis, a fixed design was used, encompassing the nominal effective concentrations (ECs) for LSM obtained from the dose-range finding experiments. The following endpoints were selected: for single chemicals, EC10, EC25, EC50 (Table 1); mixtures, ^Ni^EC10+^CHP^EC10 and ^Ni^EC25+^CHP^EC25. Mussels were exposed for 4 days as previously indicated. After treatments, digestive glands from female sex specimens were rapidly removed and washed in artificial seawater buffered with 20 mM Hepes pH 7.4 and stored according to further analysis. For transcriptomics, the tissue was kept at -20°C in a RNA preserving solution (RNA Later, Sigma-Aldrich); for histochemistry the digestive gland of 5 animals sampled from each of the five vessels was mounted on aluminum chucks and frozen in super-cooled n-hexan as previously described [69]. Two different chucks were prepared per group of mussels. The rest of the tissue, once excised, was snap-frozen in liquid nitrogen and stored at -80°C until analysis.

For toxicokinetics assessment, mussels (30 individuals per condition) were exposed for 4 days to nickel or Chlorpyrifos at the nominal EC50 for effects on LMS and to ^Ni^EC25+^CHP^EC25 in the case of the mixture. After the intoxication period, animals were transferred to clean seawater for 6 days. Sampling of test animals was performed at 3, 6, 12, 24 hours, 3 and 6 days. One vessel per condition, each containing 30 mussels was set up.

### Lysosomal responses

Cryostat sections (10 μm) were obtained through a Leica cryostat apparatus at -27°C. LSM was evaluated using the method described by [69]. Staining intensity of lysosomes was obtained by means of an inverted Axiovert microscope (Zeiss) at 400×magnification, connected to a digital camera (Axiocam, Zeiss). Digital image analysis was carried out using the Scion Image software package (Scion Corp. Inc.) from 8-bit gray scale images.

The lipid content was assessed by staining tissue sections with the oil-soluble dye, Oil Red-O (ORO) as indicated by [39] and quantified by digital image analysis as described above.

### Acetylcholinesterase activity determination

Acetylcholinesterase (AChE) activity in gill extracts was determined using the Acetylcholinesterase Reagent Kit (Ikzus) using 2 mM acetylthiocholine as substrate, essentially as described in [36]. For each condition, pools of gill portions obtained from 6 animals sampled from each of the five vessels were used for the analysis.

### Microarray analysis

Dual color competitive hybridizations were carried out following a common reference design in which each experimental condition was hybridized against the same reference condition, i.e. digestive gland tissue from vehicle-treated animals. Five different biological replicates were obtained from each of the five vessels and further used to analyze each condition, with the exception of CHP for which seven samples were analyzed in an attempt to obtain more differentially expressed genes (negligible differences were obtained using five or seven arrays, data not shown). One replicate per array was used. Total RNA was extracted from pools of 6 digestive gland pieces by the acid phenol-chloroform procedure according to [70], using the TRI-Reagent (Sigma-Aldrich). RNA was further purified by precipitation in the presence of 1.5 M LiCl_2_. The quality of each RNA preparation was verified both by UV spectroscopy and TBE agarose gel electrophoresis, in the presence of formamide as described by [19]. Competitive dual-color microarray hybridization analysis was performed using the Mytarray V1.0 and V1.1 platform [15,67]. This array encompasses 3' cDNA probes representing 1748 independent mussel sequences obtained from unbiased *M. galloprovincialis *tissues-specific normalized cDNA libraries. cDNA fluorescence-labeled probes were obtained by the direct labeling procedure in the presence of modified cy-3 and cy5 dCTP (Perkin Elmer) [67]. The procedure was carried out from 15 μg total RNA essentially as described by [19] with the exception that first strand synthesis was carried out with 0.5 μg of an anchored oligo dT(19)VN instead of random examers. Microarray slides pre-hybridised with the formamide-based buffer Northern Max (Ambion) for 1-2 h at 42°C were further hybridized overnight at 42°C with cDNA probes resuspended in 20 μl of the same buffer. After hybridisation, slides were washed to remove excess probes and unspecific binding. Three washing steps were carried out: first washing in 1× SSC, 0.2% SDS for 5 min; second washing in 0.1× SSC, 0.2% SDS for 3 min; third washing in 0.1×SSC for 5 min, the last repeated once for 3 min. All washing steps were performed with gentle shaking at room temperature. Laser scanning of microarrays was performed using a ChipReader at 5 micron resolution (Bio-Rad Laboratories, CA, USA). 16 bit TIFF images were analyzed by means of Genepix 6.0 (Axon) to get raw fluorescence data from each spot. Pre-processing and differentially expressed genes were obtained by means of an R-based package LIMMA [71] through the implementation of empirical Bayes statistics. B > 0 was used, where B-statistics represents the log-odds that a particular gene is differentially expressed. Microarray experiments were deposited in the Gene Expression Omnibus (GEO) database with the Series record [GSE21229].

### QPCR analysis

Q-PCR analysis was carried out from 25 ng RNA reverse-transcribed cDNA obtained from the same pools used for microarray hybridization. Reverse transcription was performed from 1 μg total RNA according to [72]. Relative expression levels of the following genes, actin [GeneBank:AJ625116], GM2-AP [GeneBank:AJ624495, GeneBank:AJ624405], hexosaminidase [GeneBank:AJ623463], apolipophorin precursor [GeneBank:AJ625863], chitinase [GeneBank:AJ624093, GeneBank:AJ625569, GeneBank:AJ624637, GeneBank:AJ625051] were expressed as group mean and geometrically normalized on 18S rRNA (L33452) and an invariant alkaline phosphatase gene [Geneank:AJ626187]. Genes of interest were amplified into a CFX384 quantitative thermal cycler (Bio-Rad) in triplex TaqMan assay in the presence either of the "No Rox Multiplex Quantitect" (Qiagen) or "iQTM Multiplex Powermix" (Bio-Rad) master mix. In all cases except one, it was followed the protocol for triplex mode with an annealing temperature of 60°C. In the case of the chitinase gene [GeneBank:AJ625051] the annealing temperature was selected through a preliminary gradient run and further set for the quantitative analysis at 47.7°C. Reference genes were run in a duplex assay. To this aim, 0.25 ng RNA reverse-transcribed to cDNA was amplified in the presence of 0.1 μM each dual labelled probe (Texas Red/BH2 for 18S rRNA; HEX/BH1 for alkaline phosphatase), 0.1 μM and 0.4 μM each forward and reverse primer pairs, respectively for 18S rRNA and alkaline phosphatase. The thermal protocol using the iQTM Multiplex Powermix" (Bio-Rad) was as follows: 30 sec at 95°C, followed by 40 cycles (10 s at 95°C, 20 s at 60°C) into a CFX 384 Bio-Rad PCR apparatus. All Q-PCR reactions were performed on four biological replicates and three technical replicates. Primers and probes -designed by means of Beacon Designer V. 3.0 software (Premier Biosoft International, Inc.) - are reported in Additional file 5. Metallothionein (mt10 and mt20) mRNA levels were evaluated as previously described [72]. The p53-like mRNA level [Genebank:AJ625243] was evaluated as previously reported [73]. All statistical computation and analysis of Q-PCR data were carried out using the REST and REST-mcs software [74].

### Functional genomic analysis

The functional characterization of mussel genes present in the array was based on Gene Ontology annotation and was carried out by means of the universal platform Blast2GO (B2GO) [40] using default parameters. GO term enrichment analysis was carried out through the implementation of a hypergeometric statistic (p < 0.05) [75].

### Chemical analysis

Nickel was determined in mussel tissues (about 0.5 g of a 1:1 homogenate in double distilled water) by inductively coupled plasma-mass spectrometry (ICP-MS). Samples were added of 5 ml of concentrated 65% nitric acid and introduced into a microwave oven for the mineralization. The sample was then filtered on a nitrocellulose membrane (0.45 μm) and the Ni quantified using a VG Plasma Quad 3 (VG Elemental) Inductively Couple Plasma (ICP) Mass Spectrometer. Procedure validation was performed using the Std CRM 145 R reference material containing added known amounts of metal [76]. Chlorpyrifos-ethyl residual analysis was performed in the tissue of mussels (10 g) by means of homogenization in the presence of anhydrous NaSO_4 _(10 g), dichloromethane Soxhlet extraction (5 h, with reflux rate 3-5 min), concentration in rotavapor (T = 55°), and further Gas Chromatography Mass Spectrometry, equipped with an Electron Capture Detector (ECD). GC conditions were as follow: Splitless injector (splitless time: 1 min), T = 250°C; Carrier: The constant flux at 1 ml/min; Temperature gradient: 100°C 1 min; 5°C/min up to 250°C for 1 min; 30°C/min up to 300°C for 10 min. Detector settings were the following: transfer line, T = 300°C; Source, T = 200°C. Chlorpyrifo-ethyl ion was detected at 314 m/z. Internal and external calibration procedures were performed. The analytical measurements were carried out in triplicate from pools of 4-5 animals.

### Data modeling and statistics

Biomarkers data where analyzed with the Mann Whitney U-test (n = 10). Log-logistic regression curves describing dose-dependent effects for LMS were obtained using the software package Sigma Plot 9 (Systat Inc.). The health status of mussels has been determined applying an expert system for classification able to rank the stress syndrome evolution by integrating the results from a battery of biomarkers [38]. Results on pollutant-exposed organisms are compared with those obtained from control animals, applying the non-parametric statistical tests Mann-Whitney U-test. Significant changes (p < 0.05) are converted into alteration levels (ALs) by comparison with specific thresholds. Finally data are integrated into a health status index (HSI), ranging from A (healthy) to E (pathologically stressed). HSI levels are calculated integrating ALs with a classification algorithm based on rules in the "if...then..." form: synthetically, HSI depends on the number of altered biomarkers and on the level of biological organization affected by pollutants (i.e. cell, tissue, organism) [38].

Statistically significant deviations of biomarkers responses from mixture toxicity reference models were analyzed using the MIXTOX spreadsheet model developed by [7]. Briefly, the MIXTOX software allows deviations from either the Concentration Addition [4] or Independent Action [5] model to be significance tested and analyzed in detail using a response surface analysis framework designed to identify biologically relevant response patterns like overall synergy, antagonism as well as more complex issues of ration and effect level dependent deviations.

The toxicokinetic models were computed using Sigma SYSTAT 10 from the following equation [77].

C_t _= C_0 _· e ^-kt^

Where *C*_*t *_is the pollutant concentration in an animal at time t, *C*_0 _is the pollutant concentration at the beginning of the detoxification phase and *k *is the elimination rate constant.

## Authors' contributions

FD conceived of the study, and participated in its design and coordination and drafting of the manuscript. MB carried out microarray hybridization analysis and participated in the drafting of the manuscript. AN, participated in microarray analysis, carried out Q-PCR analysis and participated in the drafting of manuscript. LB, carried out the exposure of animals, and part of biomarker analysis. AD carried out statistics of biological data and expert system analysis. AV conceived of the study, and participated in its design and coordination. All authors read and approved the final draft.

## Supplementary Material

Additional file 1 **Dose response effects of single chemicals on digestive gland lysosomal membrane stability**. Click here for file

Additional file 2 **Microarray data**: expression values and statistics. Click here for file

Additional file 3 **GO term over-representation analysis**. Click here for file

Additional file 4 **Supplementary file 3**. Additional information to Figure 6. GO terms used to generate multi level pie charts shown in Figure 6. Click here for file

Additional file 5 **Sequences of Q-PCR primers and probes**. Click here for file
