# Supplementary material for: Interactions of a pesticide/heavy metal mixture in marine bivalves: a transcriptomic assessment
Source: BMC Genomics. 2011 Apr 16;12:195. doi: 10.1186/1471-2164-12-195 (PMC3094310; doi:10.1186/1471-2164-12-195)
Supplement: Additional file 1 — Dose response effects of single chemicals on digestive gland lysosomal membrane stability. [file 1471-2164-12-195-S1.PDF]

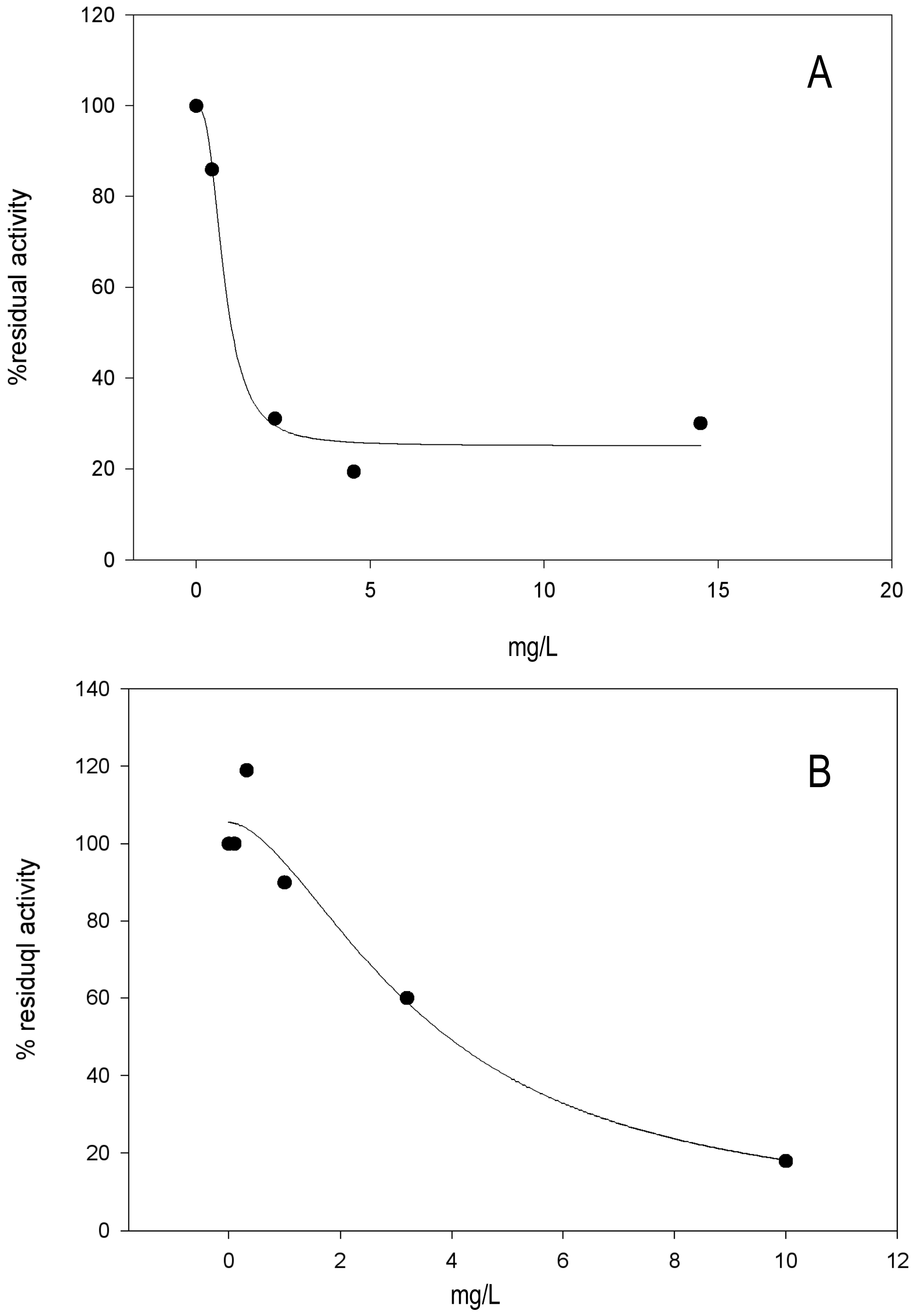

Dose response effects of single chemicals on mussel digestive gland on digestive gland lysosomal membrane stability. (log logistic regression). Panel A: Ni; Panel B: Chlorpyrifos-ethyl. 100% activity corresponded to 31.8 min N-acetyl-β-hexosaminidase latency in acid buffer destabilized lysosomes. The analysis were carried out on frozen cryostat sections according to the method of Moore [39]. (n=10)
