# Supplementary material for: Interactions of a pesticide/heavy metal mixture in marine bivalves: a transcriptomic assessment
Source: BMC Genomics. 2011 Apr 16;12:195. doi: 10.1186/1471-2164-12-195 (PMC3094310; doi:10.1186/1471-2164-12-195)
Supplement: Additional file 2 — Microarray data: expression values and statistics. [file 1471-2164-12-195-S2.PDF]

Additional file 2. Microarray gene expression values

			Ni		Chp		Mix	
Array_ID	*EMBL*	*B2GO annotation*	*M*	*B*	*M*	*B*	*M*	*B*
Myt01-004F11	AJ623426	---NA---	-2.17	16.3	-0.45	-2.3	-0.13	-4.9
Myt01-017B11	AJ625979	defender against cell death 1	-1.95	14.8	-0.21	-5.9	0.04	-6.5
Myt01-004F08	AJ623418	---NA---	-2.27	13.4	-1	-0.4	-0.24	-4.1
Myt01-005B04	AJ623522	---NA---	-2.38	12.3	-0.42	-4.0	-0.38	-1.0
Myt01-016C08	AJ625847	metallothionein isoform mt-10b	1.46	11.5	-0.13	-6.3	0.89	4.4
Myt01-001E12	AJ516452	ependymin-related protein	2.38	11.2	-0.37	-6.2	0.49	-1.6
Myt01-001B07	AJ516390	muc2 protein	1.05	11.0	0.19	-5.1	-0.01	-6.5
Myt01-017B12	AJ625981	developmentally-regulated vdg3	0.86	10.3	-0.32	-3.5	-0.09	-5.9
Myt01-009E10	AJ624495	gm2 ganglioside activator protein	-0.87	9.7	-0.61	-5.0	0.36	0.1
Myt01-004H03	AJ623460	gram negative bacteria binding protein 2	-0.79	7.9	-0.47	0.7	-0.25	-1.7
Myt01-008H08	AJ624368	scavenger receptor cysteine-richpartial	-1.05	7.8	-1.48	6.1	0.46	0.2
Myt01-005F12	AJ623632	---NA---	-0.81	7.6	1.61	1.8	1.02	0.3
Myt01-012D02	AJ625051	chitinase 1	-0.80	7.5	3.17	11.5	2.01	0.3
Myt01-009A02	AJ624380	loc553472 protein	-0.57	7.4	-0.08	-6.4	0.17	-4.9
Myt01-004C02	AJ623315	glycosidefamily 9	-0.91	7.3	-0.43	-5.3	0.61	2.0
Myt01-009A12	AJ624405	gm2 ganglioside activator protein	2.63	7.0	0.46	-5.4	0.46	-1.2
Myt01-017D03	AJ626007	---NA---	-0.66	6.8	-0.25	-5.5	0.12	-5.8
Myt01-009F02	AJ624501	---NA---	2.01	6.3	-0.39	-4.3	-0.6	-0.8
Myt01-009A05	AJ624383	b-cell translocation geneanti-proliferative	0.63	6.3	-0.02	-6.5	-0.25	-3.6
Myt01-013H05	AJ625350	---NA---	-0.88	6.3	2.32	20.6	1.36	1.8
Myt01-004F09	AJ623424	connective tissue growth factor	-0.67	6.2	-1.06	7.1	-0.78	-1.3
Myt01-009B04	AJ624414	gi|118195|sp|P01038|CYT_CHICK Cystatin precursor (Egg-white cystatin)	-0.47	6.2	-0.53	0.1	0.44	0.9
Myt01-002D03	AJ516600	h3family 3b	-0.77	6.1	0.39	-4.2	0.29	-3.4
Myt01-016C10	AJ625851	developmentally-regulated vdg3	0.58	5.9	-0.49	-0.5	0.2	-3.6
Myt01-004D02	AJ623337	---NA---	-0.64	5.9	-0.1	-6.4	0.38	-0.8
Myt01-005C12	AJ623552	dbh-like 1	-0.83	5.6	-0.41	-5.6	0.2	-5.8
Myt01-011F02	AJ624906	gi|342490|gb|M83761.1|MSLMTFR6A Mytilus edulis mitochondrial NADH	-0.59	5.4	0.05	-6.5	-0.22	-4.4

		dehydrogenase subunit 2 (ND2)						
Myt01-001H12	AJ516519	adp-ribosylation factor 1	-0.50	5.4	-0.08	-6.5	0.15	-5.2
Myt01-002B12	AJ516568	---NA---	-0.55	5.4	0.61	0.0	0.1	-5.7
Myt01-012B07	AJ625004	---NA---	0.58	5.4	-0.78	-5.6	-0.36	-1.7
Myt01-011G05	AJ624926	small heat shock protein p26	0.63	5.3	-0.12	-6.5	0.09	-6.4
Myt01-016B08	AJ625829	gram negative bacteria binding protein 2	-0.86	5.3	-0.32	-3.7	-0.3	-2.5
Myt01-012F06	AJ625109	---NA---	-0.44	5.2	0.19	-6.3	0.18	-4.2
Myt01-016D06	AJ625863	apolipophorin precursor	0.56	5.1	-0.12	-6.1	-0.05	-6.4
Myt01-009F07	AJ624511	---NA---	0.74	5.0	-0.49	-4.1	-0.39	-3.7
Myt01-005D07	AJ623567	---NA---	0.59	4.9	-0.11	-6.3	0.37	0.3
Myt01-011D05	AJ624869	cathepsin l	0.84	4.9	-0.55	-2.4	0.68	4.1
Myt01-002A11	AJ516537	small nuclear ribonucleoprotein b	-0.59	4.7	0.24	-6.2	0.44	-4.2
Myt01-019B10	AY484747	mitochondrial nadh dehydrogenase ND1	-0.50	4.7	-0.19	-6.1	-0.26	-5.1
Myt01-009A10	AJ624395	fatty acid binding proteinbrain	0.61	4.5	-0.08	-6.5	-0.17	-5.0
Myt01-005B01	AJ623517	mantle gene 8	0.60	4.2	0.31	-5.3	0.41	-2.1
Myt01-005G02	AJ623636	---NA---	-1.20	4.2	2.2	5.6	1.8	1.2
Myt01-002E09	AJ516628	heterochromatin protein 1 beta	-0.69	4.1	0.23	-6.3	-0.46	-1.4
Myt01-009B02	AJ624410	---NA---	0.43	4.1	-0.25	-5.0	0.39	0.3
Myt01-016H12	AJ625935	---NA---	0.50	4.0	0.23	-5.5	0.08	-6.3
Myt01-010C06	AJ624643	---NA---	-0.57	4.0	1.8	6.1	1.02	1.5
Myt01-013C04	AJ625226	galactoside-8 (galectin 8)	-0.44	3.9	-0.26	-6.2	-0.01	-6.6
Myt01-012A08	AJ624979	---NA---	-0.51	3.8	-0.46	-6.0	-0.55	-2.3
Myt01-004G10	AJ623452	---NA---	0.44	3.8	-0.39	-2.7	0.4	0.5
Myt01-009E08	AJ624492	---NA---	-0.53	3.8	-0.51	-2.2	-0.38	-1.4
Myt01-002F06	AJ516643	tubulin-specific chaperone a	0.41	3.7	0.04	-6.5	-0.19	-4.6
Myt01-015E06	AJ625661	atp synthase beta subunit	-0.39	3.7	-0.24	-5.0	-0.18	-4.0
Myt01-004E09	AJ623395	---NA---	-0.52	3.6	-0.17	-6.2	0.13	-5.3
Myt01-016G08	AJ625914	vitelline envelope zona pellucida domain 9	-0.56	3.6	0.58	2.9	0.31	-3.9
Myt01-010C02	AJ624637	chitinase	-0.52	3.6	1.58	4.5	1.68	1.8
Myt01-005B10	AJ623529	---NA---	-0.34	3.5	0.11	-6.4	-0.11	-5.9

Myt01-005G07	AJ623650	serpine1 mrna binding protein 1	-0.74	3.4	0.24	-6.4	-0.01	-6.5
Myt01-012E08	AJ625083	small nuclear ribonucleoprotein d2	-0.59	3.4	0.35	-4.2	0.12	-5.7
Myt01-010H09	AJ624775	mitochondrial aldehyde dehydrogenase 2	-0.52	3.2	-0.47	-4.7	-0.02	-6.6
Myt01-016E02	AJ625872	vitelline envelope zona pellucida domain 7	-0.46	3.1	0.57	-5.7	-0.07	-6.4
Myt01-010H04	AJ624759	---NA---	0.65	3.1	0.04	-6.5	0	-6.6
Myt01-002B04	AJ516549	---NA---	-0.58	3.0	0.13	-6.3	0.46	-0.4
Myt01-012E06	AJ625081	hydroxysteroid dehydrogenase like 2	0.39	3.0	-0.01	-6.6	0.13	-4.8
Myt01-016B10	AJ625831	---NA---	0.29	3.0	-0.03	-6.5	0.03	-6.5
Myt01-017H04	AJ626179	small nuclear ribonucleoprotein sm d1	-0.54	2.9	0.31	-4.9	0.16	-4.4
Myt01-015G10	AJ625734	---NA---	-1.09	2.8	1.48	2.2	0.92	-0.8
Myt01-006F12	AJ623853	---NA---	-0.45	2.7	-0.3	-5.2	0.1	-6.0
Myt01-002G07	AJ516663	h2a histonemember z	-0.76	2.7	0.65	-3.1	0.08	-6.3
Myt01-009D11	AJ624472	---NA---	0.68	2.6	-0.56	-2.7	0.32	-1.6
Myt01-005D09	AJ623570	polybindingcytoplasmic 4 (inducible form)	-0.47	2.6	0.09	-6.4	0.2	-4.7
Myt01-010H07	AJ624770	---NA---	0.62	2.6	-0.19	-6.2	0.15	-5.7
Myt01-007A02	AJ623940	---NA---	-0.99	2.6	1.93	8.5	0.99	-0.3
Myt01-014G04	AJ625502	---NA---	0.38	2.6	-0.01	-6.5	-0.17	-5.3
Myt01-004G02	AJ623429	developmentally-regulated vdg3	0.52	2.4	-0.36	-3.4	0.07	-6.4
Myt01-016F03	AJ625893	---NA---	-0.30	2.4	0.43	1.7	0.05	-6.4
Myt01-011D12	AJ624886	---NA---	0.48	2.4	0.17	-6.2	-0.19	-5.1
Myt01-007F12	AJ624093	chitinase	-0.41	2.3	1.92	9.9	2.02	-0.4
Myt01-014B01	AJ625383	ribosomal protein l37a	-0.45	2.1	0.07	-6.5	0.09	-5.8
Myt01-013E08	AJ625276	---NA---	-0.83	2.1	1.85	3.4	1.58	-0.9
Myt01-010C07	AJ624645	prohibitin	-0.55	2.1	-0.02	-6.6	-0.05	-6.5
Myt01-002B03	AJ516547	cytoplasmic intermediate filament protein	0.29	2.0	-0.04	-6.5	0.09	-5.7
Myt01-001C02	AJ516404	small nuclear ribonucleoprotein d3	-0.46	2.0	-0.01	-6.5	0.15	-5.5
Myt01-013C10	AJ625241	---NA---	0.48	2.0	-0.01	-6.6	-0.23	-4.5
Myt01-010H01	AJ624755	---NA---	0.53	2.0	-0.18	-6.4	0.14	-6.1
Myt01-001B01	AJ516372	elastin microfibril interfacer 2	-0.43	1.9	0.03	-6.5	0.55	0.1
Myt01-006B07	AJ623719	eif3s10 protein	-0.35	1.9	-0.3	-5.5	-0.04	-6.5

Myt01-019B02	AY484747	mItochondrial nadh dehydrogenase ND1	0.37	1.8	0.26	-4.1	0.08	-6.1
Myt01-006A06	AJ623696	---NA---	0.30	1.7	0.87	4.8	0.76	-2.3
Myt01-002D04	AJ516601	---NA---	-0.62	1.6	-0.06	-6.5	-0.13	-6.1
Myt01-005E10	AJ623603	---NA---	0.50	1.5	-0.39	-2.8	0.1	-5.9
Myt01-013A12	AJ625189	---NA---	0.37	1.5	-0.23	-6.3	-0.4	-5.0
Myt01-005D10	AJ623571	---NA---	-0.35	1.5	-0.28	-4.1	0.61	-0.7
Myt01-003E11	AJ516802	ependymin related protein 1	0.36	1.4	-0.53	-3.7	0.2	-3.3
Myt01-001B09	AJ516392	ribosomal protein l28	-0.48	1.4	-0.23	-5.7	-0.31	-4.2
Myt01-009A08	AJ624392	epididymal secretory protein e1 precursor (niemann pick type c2 protein homolog) (kda secretory protein)	-0.45	1.3	0.38	-5.8	0.74	-0.5
Myt01-009G12	AJ624549	---NA---	-0.26	1.3	0.05	-6.5	-0.17	-5.5
Myt01-017H09	AJ626199	ribosomal protein l23a	0.56	1.3	-0.36	-4.9	-0.42	-4.9
Myt01-017E01	AJ626029	gi|2541910|dbj|BAA22850.1| troponin T [Mizuhopecten yessoensis]	-0.34	1.3	0.61	-4.5	0.41	-3.3
Myt01-014B03	AJ625387	ekn1	0.30	1.2	0.08	-6.5	-0.17	-5.2
Myt01-017A09	AJ625954	---NA---	0.41	1.2	-0.04	-6.5	-0.18	-5.5
Myt01-006C06	AJ623739	---NA---	-0.42	1.2	1.82	11.6	2.45	3.7
Myt01-010G03	AJ624733	mantle gene 8	0.52	1.0	0.07	-6.5	0.33	-2.0
Myt01-006C03	AJ623735	integrin, alpha d precursor	-0.31	0.9	-0.04	-6.5	0.36	2.5
Myt01-007B10	AJ623981	---NA---	-0.32	0.9	-0.25	-5.9	0.26	-3.7
Myt01-016H11	AJ625934	ribosomal protein s15a	-0.27	0.9	-0.08	-6.3	0.07	-6.1
Myt01-009D08	AJ624465	cg3355-isoform a	0.25	0.8	-0.41	-1.4	0.27	-3.4
Myt01-008C07	AJ624248	elongation factor 2	-0.65	0.8	-0.15	-6.1	-0.26	-1.4
Myt01-006F05	AJ623834	---NA---	-0.31	0.8	-0.1	-6.4	-0.05	-6.3
Myt01-013G01	Myt01-013G01	---NA---	-0.89	0.7	1.52	-3.5	0.72	-2.8
Myt01-001F12	AJ516476	---NA---	0.29	0.7	0.1	-6.1	-0.28	0.7
Myt01-012H02	AJ625145	ribosomal protein l22	-0.38	0.7	0.05	-6.5	-0.28	-3.9
Myt01-006F07	AJ623838	---NA---	-0.36	0.7	0.16	-6.4	0.24	-3.8
Myt01-003A09	AJ516699	gi|2353340|gb|AAB69448.1| Tis11 [C. virginica]	-0.38	0.6	-0.4	-5.9	-0.05	-6.4
Myt01-016H02	AJ625922	---NA---	-0.29	0.6	0.42	-6.3	-0.44	-4.5

Myt01-014B09	AJ625400	---NA---	-0.48	0.6	0.12	-6.5	0.16	-5.9
Myt01-018G05	AJ626409	---NA---	-0.36	0.6	-0.19	-6.3	-0.2	-5.3
Myt01-011C11	AJ624846	---NA---	-0.38	0.5	0.41	-4.7	0.02	-6.5
Myt01-017E10	AJ626060	---NA---	0.30	0.5	0.04	-6.5	0.08	-5.8
Myt01-008H05	AJ624361	matrilin-4-like protein	0.35	0.5	0.46	-6.0	0.35	-5.4
Myt01-008H03	AJ624358	---NA---	0.89	0.5	-0.13	-6.4	-0.1	-6.1
Myt01-016G12	AJ625919	---NA---	0.26	0.5	-0.04	-6.5	0.09	-6.0
Myt01-003F03	AJ516810	---NA---	0.34	0.4	0.14	-6.1	0.01	-6.5
Myt01-009B06	AJ624418	myc homolog	-0.26	0.4	-0.51	-3.3	-0.46	-2.5
Myt01-005E01	AJ623586	---NA---	0.62	0.4	-0.14	-6.5	-0.51	-0.5
Myt01-004H09	AJ623481	ependymin related protein 1	0.34	0.3	-0.12	-6.4	0.41	-0.8
Myt01-009A07	AJ624391	---NA---	-0.51	0.2	-0.11	-6.5	0.07	-6.3
Myt01-009E03	AJ624481	endo-beta--glucanase	-0.38	0.2	-0.12	-6.4	0.03	-6.5
Myt01-002C05	AJ516582	h2a histonemember z	-0.31	0.2	0.08	-6.5	0.56	1.2
Myt01-012C10	AJ625039	---NA---	0.49	0.2	-0.36	-5.6	-0.23	-4.9
Myt01-012A04	AJ624969	fk506-binding protein	-0.38	0.1	0.39	-5.7	0.08	-6.4
Myt01-011E01	AJ624887	---NA---	-0.31	0.1	0.36	-5.3	-0.19	-6.2
Myt01-010B01	AJ624607	Collagen type-alpha 1	-0.43	0.1	-0.02	-6.6	0.14	-5.2
Myt01-016H06	AJ625926	dopamine beta hydroxylase-like protein	0.28	0.1	-0.15	-6.0	0.04	-6.5
Myt01-015A06	AJ625549	ribosomal protein l27a	-0.45	0.1	-0.25	-4.9	0.09	-5.9
Myt01-018E05	AJ626346	---NA---	-0.25	0.1	0.18	-6.3	-0.25	-2.1
Myt01-002C03	AJ516578	cathepsin l	0.24	0.1	-0.38	-3.4	0.19	-3.4
Myt01-008C04	AJ624236	---NA---	0.56	0.0	-0.59	-0.8	-0.25	-3.5
Myt01-005D06	AJ623565	gi|102882|pir||C41711 defensin C - beetle (Zophobas atratus)	-0.69	0.0	-0.12	-6.3	0.11	-5.5
Myt01-018F06	AJ626388	histone aminotransferase 1	-0.55	0.0	0.04	-6.5	-0.8	-2.7
Myt01-005D11	AJ623579	sarcoplasmic calcium-binding protein	-0.44	-0.2	1.08	4.4	0.42	-3.3
Myt01-009F06	AJ624509	phospholipasegroup xiia	-0.24	-0.6	-0.06	-6.4	0.34	2.0
Myt01-002G01	AJ516656	sialic acid acetylesterase	-0.24	-0.7	-0.28	-6.2	-0.97	0.0
Myt01-003E07	AJ516796	beta tubulin	0.25	-0.7	0.11	-6.2	0.5	3.2
Myt01-008H01	AJ624353	alpha 3 type vi collagen isoform 1 precursor	-0.20	-0.8	-0.7	8.8	-0.04	-6.4
Myt01-005B05	Myt01-005B05	---NA---	-0.21	-0.8	-0.13	-5.8	0.47	4.1

Myt01-014A08	AJ625375	---NA---	-0.33	-0.9	0.97	-1.7	1.28	0.8
Myt01-012C04	AJ625031	---NA---	-0.50	-1.2	-0.04	-6.6	-0.45	1.7
Myt01-010F02	AJ624702	macrophage galactose n-acetyl-galactosamine specific lectin 2	-0.27	-1.6	-0.35	-4.7	-0.44	0.1
Myt01-013D11	AJ625268	ferritin	0.18	-1.8	0.55	4.2	-0.08	-5.8
Myt01-009F03	AJ624502	mam domain containing 2	0.24	-1.8	-0.57	1.1	0.4	1.0
Myt01-009B07	AJ624419	mucin-like protein	-0.26	-2.1	-0.88	7.3	0.16	-4.8
Myt01-015H05	AJ625778	chitinase	-0.25	-2.1	0.58	-4.3	1.95	5.0
Myt01-012F02	AJ625103	chromodomain helicase dna binding protein 5	-0.44	-2.2	0.22	-5.9	-0.43	0.1
Myt01-011D07	AJ624871	ribosomal protein s25	-0.43	-2.3	-0.3	-4.7	-0.36	1.1
Myt01-005A10	AJ623510	---NA---	0.19	-2.5	0.57	2.0	0.08	-6.2
Myt01-006B03	AJ623709	---NA---	0.20	-2.9	0.09	-6.4	-0.36	0.7
Myt01-010B11	AJ624630	---NA---	0.19	-3.0	-0.27	-3.9	-0.5	1.8
Myt01-009E01	AJ624478	ribophorin ii	-0.23	-3.1	0.21	-5.8	-0.42	3.6
Myt01-006H04	AJ623902	---NA---	-0.37	-3.2	1.02	1.6	0.4	-3.6
Myt01-007F02	AJ624082	upstream of nras	-0.33	-3.5	-0.14	-6.2	-0.57	0.1
Myt01-005C05	AJ623544	ependymin related protein-1 precursor	0.18	-3.6	-0.01	-6.6	0.39	0.6
Myt01-017C08	Myt01-017C08	---NA---	0.27	-3.6	-0.01	-6.6	0.96	1.4
Myt01-004D10	AJ623352	exosome component 2	-0.15	-4.0	0.12	-6.1	-0.41	3.2
Myt01-009E07	AJ624491	trypsin-like proteinase	-0.41	-4.0	-0.33	-6.1	0.67	5.0
Myt01-015D10	AJ625641	---NA---	0.17	-4.2	-0.22	-5.2	-0.42	2.3
Myt01-017D04	AJ626008	---NA---	0.16	-4.4	0.2	-5.2	-0.37	1.6
Myt01-003D05	AJ516764	---NA---	0.61	-4.6	0.29	-5.1	-0.41	0.1
Myt01-008H06	AJ624363	mam domain containing 2	0.11	-4.8	-0.48	0.3	0.16	-5.1
Myt01-015G02	AJ625706	---NA---	0.20	-4.9	-0.24	-5.3	-0.42	0.1
Myt01-013G03	AJ625320	---NA---	0.13	-4.9	0.5	-5.3	0.94	3.3
Myt01-017G04	AJ626137	---NA---	-0.33	-5.0	1.02	0.2	0.8	-1.3
Myt01-005G05	AJ623646	---NA---	0.31	-5.0	-0.39	-6.2	-0.53	0.3
Myt01-006A11	AJ623703	gi|21105298|gb|AF448524.1| Mytilus galloprovincialis precollagen-NG	-0.10	-5.4	-0.15	-5.6	-0.6	4.6
Myt01-010A03	AJ624584	cytochrome b	0.17	-5.5	0.58	2.2	0.17	-5.0
Myt01-006E03	AJ623789	1-Precollagen-P	-0.13	-5.5	0.2	-6.2	0.31	1.0

Myt01-016F08	AJ625899	spi1_craviserine protease inhibitor cvsi-1 precursor	0.13	-5.5	-0.66	3.8	0.31	-1.3
Myt01-016E05	AJ625876	---NA---	-0.24	-5.6	0.01	-6.6	-0.51	4.0
Myt01-018A02	AJ626210	---NA---	-0.13	-5.6	-0.21	-6.4	0.51	2.8
Myt01-010H05	AJ624761	translationally controlled tumor protein	-0.12	-5.7	0.09	-6.4	0.3	0.9
Myt01-004B08	AJ516900	variable region-containing chitin-binding protein 3	0.10	-5.7	-0.18	-5.8	0.51	1.0
Myt01-014A02	AJ625361	elongation factor-1 alpha	-0.10	-5.7	0.18	-6.4	0.9	0.2
Myt01-002E02	AJ516619	vitelline coat lysin m7	-0.17	-5.8	0.04	-6.5	0.61	0.1
Myt01-006C05	AJ623737	growth arrest and dna-damage-gamma	0.19	-5.8	-0.01	-6.5	-0.79	0.5
Myt01-007H10	AJ624162	sam domain-containing protein	-0.12	-5.9	1.72	7.9	2.75	3.7
Myt01-007F07	AJ624087	chitinase b precursor	-0.44	-6.2	0.04	-6.5	0.78	3.6
Myt01-005B12	AJ623532	myc homolog	-0.06	-6.3	-0.31	-2.9	-0.35	0.6
Myt01-015E04	AJ625651	---NA---	0.13	-6.4	0.21	-6.5	-0.92	0.9
Myt01-008G02	AJ624336	gi|20377786|gb|AAM20843.1| AF369699_1 SHG [Littorina littorea]	0.11	-6.4	-0.52	2.2	0.02	-6.5
Myt01-001A08	AJ516362	---NA---	0.09	-6.5	-0.27	-5.9	-0.4	0.3
Myt01-012A11	AJ624990	---NA---	-0.31	-6.6	2.49	11.8	1.37	0.8
Myt01-013G07	AJ625330	---NA---	0.16	-6.7	0.26	-5.3	0.41	1.1
Myt01-018C02	AJ626279	---NA---	-0.05	-6.7	0.3	-4.2	0.67	1.3
Myt01-013D07	AJ625263	---NA---	0.16	-6.7	-0.19	-5.9	-0.32	1.2
Myt01-002F03	AJ516640	ficolin b	0.11	-6.8	-0.1	-6.5	-0.45	0.1
Myt01-009B11	AJ624428	---NA---	0.08	-6.9	-0.35	-4.3	-0.35	0.4
Myt01-014F12	AJ625490	caspase 7	-0.06	-6.9	0.12	-6.4	-0.38	0.1
Myt01-012F08	AJ625116	beta actin	-0.05	-6.9	0.07	-6.3	0.51	2.7
Myt01-017B10	AJ625974	heat shock protein 90	0.04	-7.0	0.46	2.9	-0.05	-6.2
Myt01-015D01	AJ625621	heat shock protein 90	0.06	-7.0	-0.06	-6.5	0.65	2.0
Myt01-002F01	AJ516636	cytochrome c oxidase subunit via polypeptide 1	0.07	-7.0	0.01	-6.6	0.33	0.8
Myt01-015H01	AJ625768	---NA---	-0.06	-7.0	-0.39	1.0	0.06	-6.1
Myt01-015C01	AJ625594	general transcription factor iii c 1	0.09	-7.0	0.8	-0.9	1.06	4.2
Myt01-009E12	AJ624499	cysteine rich protein 61	-0.05	-7.0	-0.32	-2.2	-0.31	1.2
Myt01-002G10	AJ516666	---NA---	0.15	-7.1	0.63	-5.2	-0.67	0.4
Myt01-001A07	AJ516361	ribosomal protein l9	0.06	-7.1	0.35	-4.1	0.39	0.7

Myt01-002G11	AJ516668	---NA---	0.09	-7.1	-0.05	-6.5	-0.73	0.5
Myt01-015H12	AJ625792	actin	-0.03	-7.1	-0.05	-6.5	0.38	1.1
Myt01-012G03	AJ625130	---NA---	-0.21	-7.1	2.03	7.0	1.18	-1.1
Myt01-006D06	AJ623767	---NA---	-0.09	-7.1	0.1	-6.5	1.16	0.8
Myt01-014E05	AJ625457	Collagen type alpha 1	-0.06	-7.1	0.27	-6.1	-0.36	1.9
Myt01-009C06	AJ624440	---NA---	0.08	-7.1	0.08	-6.4	-0.82	5.5
Myt01-002C10	AJ516589	---NA---	-0.12	-7.1	0.1	-6.5	-0.92	0.3
Myt01-004E03	AJ623376	chitinase	-0.11	-7.2	1.39	3.4	0.69	-2.5
Myt01-018F04	AJ626386	actin binding protein	0.04	-7.2	0.2	-5.9	-0.7	0.7
Myt01-001A01	AJ516347	---NA---	-0.11	-7.2	-0.22	-6.2	0.59	2.0
Myt01-004G03	AJ623431	---NA---	0.16	-7.2	0.02	-6.5	-0.36	0.8
Myt01-015H08	AJ625788	---NA---	-0.05	-7.2	0.11	-6.3	-0.32	0.1
Myt01-015B01	AJ625569	chitinase	-0.08	-7.2	1.95	12.2	1.69	4.7
Myt01-013F05	AJ625302	cg1637-isoform c	0.06	-7.2	0.01	-6.6	-0.46	0.2
Myt01-003D11	AJ516774	vitelline coat lysin m7	0.10	-7.3	-0.23	-6.2	1.12	4.8
Myt01-015D05	AJ625629	---NA---	0.05	-7.3	-1.01	12.2	0.07	-6.3
Myt01-016A10	AJ625805	ribosomal protein l37	0.04	-7.3	0.16	-5.2	-0.35	2.2
Myt01-014G03	AJ625500	cue domain containing 1	-0.06	-7.3	0.13	-6.4	0.71	1.2
Myt01-009A11	AJ624397	cathepsin l-associated protein	0.02	-7.4	0.44	-4.2	0.45	4.9
Myt01-010D01	AJ624658	---NA---	0.02	-7.4	0.01	-6.5	-0.45	3.0
Myt01-006G05	AJ623872	---NA---	-0.03	-7.4	0.08	-6.4	-0.39	0.1
Myt01-016A05	AJ625799	fibrinogen c domain containing 1	-0.03	-7.4	0.5	0.7	0.01	-6.6
Myt01-017G06	AJ626142	---NA---	0.02	-7.4	0.18	-5.5	-0.4	1.3
Myt01-015G11	AJ625758	---NA---	-0.02	-7.4	0.06	-6.5	0.34	0.1
Myt01-011F09	Myt01-011F09	gi|30040349|gb|AE016980.1| Shigella flexneri 2a str. 2457T section 3 of 16 of the complete genome	-0.03	-7.4	0.6	-2.9	1.02	2.8
Myt01-004H06	AJ623463	beta-n-acetylhexosaminidase	0.03	-7.5	-0.32	-6.1	1.12	-5.7
Myt01-004G09	AJ623451	---NA---	-0.02	-7.5	-0.64	0.3	-0.15	-4.2
Myt01-005A11	AJ623513	---NA---	-0.01	-7.5	0.23	-4.8	-0.31	0.5
Myt01-004A02	AJ516864	---NA---	-0.01	-7.5	-0.08	-6.5	-0.99	0.1
Myt01-001G04	AJ516482	arginine kinase	0.00	-7.5	-0.03	-6.5	1.27	6.9
Myt01-006D02	AJ623757	---NA---	0.00	-7.5	-0.05	-6.4	0.39	0.4
Myt01-013D02	AJ625253	variable surface lipoprotein vsp422-3	0.00	-7.5	0.46	-5.4	1.33	0.5

Myt01-016G04	AJ625909	---NA---	0.00	-7.5	-0.03	-6.5	-0.69	0.1

Shown are Array_ID, gene identification; EMBL, sequence accession numbers to the EMBL database; B2GO description, gene annotation from the Blast2GO tool (Conesa et al., 2005); q-PCR, log2 relative expression values obtained from q-PCR analysis (see Table 6 for details); M, log2 relative expression level obtained from microarray analysis; B, moderated B-statistics values. Microarray data were analyzed through the Linear Modes for Microarray Analysis R-based package (Smyth, 2004) and account for at least four biological and two technical replicates. The statistical threshold used for microarray analysis was B > 0 (negative B values are depicted in red for an a glance overview of significant genes). All relative expression values are referred versus a set of reference not-exposed individual mussels.
