# Supplementary material for: Interactions of a pesticide/heavy metal mixture in marine bivalves: a transcriptomic assessment
Source: BMC Genomics. 2011 Apr 16;12:195. doi: 10.1186/1471-2164-12-195 (PMC3094310; doi:10.1186/1471-2164-12-195)
Supplement: Additional file 5 — Sequences of Q-PCR primers and probes. [file 1471-2164-12-195-S5.PDF]

Additional file 5. Q-PCR primers and Taqman probes

Gene _ID	Sense Primer	Antisense Primer	Probe
L33452	CGGAGAGGGAGCATGAGAAAC	CGTGCCAGGAGTGGGTAATTT	CCACATCCAAGGAAGGCAGCAGGC
AJ625116	GTGTGATGTCGATATCCGTAAGGA	GCTTGGAGCAAGTGCTGTGA	ACGCCAACACCGTCTTGTCTGGTGG
AJ624495	AGCGGACAGTCACTACAGATT	TAAACGCATGTTCCAACTTTACG	ACATGGAACAGCGTGCCAGTTACCA
AJ624405	CAACAACACATGTCCACAGTCA	CCCACTGGGATGGAATCGTT	TCACTGTTACGACTGCGCTCTGGACA
AJ623463	CCAACAAATCCTTCCTTAGTACT	ACAGACATTCCACCAAACTGG	TCTCAGCAGACGGACTAAATGCACGT
AJ625863	TACGATGATATGATCATGCCAAA	ACATCTATCTTGAACCTGCCAT	CATCCGACGCTTCCGCTCTAGGAA
AJ624093	ACACATGGAGCATTCTATGAAGAT	GCATGTTGTTATTGCTTGTTTCC	ACTAGACCCGCAGGACATGGCATTT
AJ625569	GATTGCCGAGTTACTGCTGAT	TACAGTTTTCGTACTGAACGGAA	TGTAACCACAACACTCAGAGAGACACC
AJ624637	AACTAAGCAAACTGGTAAATGTGG	ACAGTGATAGTATCCGTTCTTGTC	CCAACGCCATGTGAACACGGAGCA
AJ625051	AACATGCGACTGGCCATAA	TTATTATTCTGTACAAAAG	AATACTTGACTCTGGAATATTAGACTGA
AJ626187	CCAGGGATACCTACTTACTGCTA	GTGCCCTCATTCCATCTGAAA	TCGTATCCGTCATTCGCCAAGGTCCA

All sequences are given in 5'-3' direction
